# Time to act: climate change and its impacts on women's sexual and reproductive health

**DOI:** 10.61622/rbgo/2025rbgoedt05

**Published:** 2025-11-18

**Authors:** Fernanda Garanhani Surita, Odette Del Risco Sánchez, Júnia Aparecida Laia da Mata

**Affiliations:** 1 Universidade Estadual de Campinas Campinas SP Brasil Universidade Estadual de Campinas, Campinas, SP, Brasil.; 2 Universidade Federal do Rio Grande do Sul Porto Alegre RS Brasil Universidade Federal do Rio Grande do Sul, Porto Alegre, RS, Brasil.

Climate change constitutes a global threat to health, as it broadly affects aspects of natural and human systems, compromising physical, social, and economic conditions, as well as the functioning of health systems.^(1)^ Its effects impact the essential components that support life, such as air, food, water, shelter, and safety.^(2)^

As climatic conditions change, extreme weather events, such as storms, heat waves, floods, droughts, and forest fires, become more frequent and intense. These affect human health directly and indirectly, increasing the risk of mortality, worsening noncommunicable diseases, causing the emergence and spread of infectious diseases, and increasing the occurrence of health emergencies. Climate shocks and growing environmental stress compromise the environmental and social determinants of physical and mental health.^(1)^

In the face of the global climate crisis, disasters have occurred in different parts of the planet. They result from adverse events, of natural origin or induced by human action, affecting ecosystems and vulnerable populations and triggering significant human, material, or environmental damage, as well as economic and social losses.^(3)^

Developing countries have a lower capacity for adaptation and response to this scenario, especially due to infrastructure limitations. According to the Intergovernmental Panel on Climate Change Report,^(4)^ the increasing frequency of extreme events has intensified food and water insecurity, disproportionately affecting regions of Africa, Asia, Latin America, small islands, the Arctic, as well as Indigenous populations and low-income groups. The report indicates that between 3.3 and 3.6 billion people live in contexts of high climate vulnerability, with the greatest risks observed among populations facing structural conditions of inequality. Data highlight that between 2010 and 2020, mortality caused by floods, droughts, and storms was 15 times higher in regions of greater vulnerability.^(4)^

Structural inequalities cause and exacerbate the impacts arising from climate change, operating as a cycle.^(5)^ The World Health Organization (WHO) estimates that by 2050 there will be 250,000 annual deaths related to climate, due to malnutrition, malaria, diarrhea, and heat stress.^(1)^

Women are more acutely affected by climate change;^(2)^ there is an increased risk of adverse maternal and perinatal outcomes. For example, high temperatures and pollution are associated with low birth weight, preterm birth and stillbirth, gestational diabetes, and hypertensive disorders in pregnancy.^(6-11)^

In countries with limited resources, access to sexual and reproductive health, such as contraception, safe abortion, and prenatal and postpartum care, already tends to be insufficient, and in disaster situations, adverse outcomes in women's health are exacerbated.^(12)^ Exposure to climate risks and their consequences can also affect mental health, increasing stress, anxiety, and depression, and contributing to intergenerational trauma.^(11)^

In humanitarian emergencies caused by disasters related to climatic events, women are more vulnerable and require special attention.^(13)^ According to the United Nations (UN), in these situations, women and girls are disproportionately affected due to gender barriers and inequalities, facing greater loss of lives and livelihoods, longer recovery periods, and impacts on life expectancy, education, housing, health, safety, nutrition, and job stability.^(14)^

Climate change also produces indirect effects, such as civil conflicts, migrations, and forced displacement, contexts in which women tend to face barriers in accessing health services, situations of discrimination, isolation, financial limitations, food insecurity, and restrictions on access to water, factors that impact health care.^(12,15)^ There is a significant increase in the vulnerabilities of girls and women to various forms of gender-based violence, including sexual violence, sexual exploitation and abuse, child marriage, human trafficking, and domestic or intimate partner violence.^(12,16,17)^ Evidence, even if underreported in these settings, indicates that approximately one in five refugee or displaced women in complex humanitarian contexts has already experienced some type of sexual violence.^(18)^

Confronting climate change is configured as a public health priority, requiring the implementation of effective actions that promote collective and individual responses to its impacts, including those related to reproductive planning.

The current scenario requires urgent actions grounded in a systemic approach to address it. Although in recent years initiatives aimed at mitigating climate impacts have been implemented on global agendas, especially through governmental commitments under the Sustainable Development Goals (SDGs), responses to the climate emergency still demand more consistent efforts, based on intersectoral cooperation and coordination among nations.

Even though they represent the most vulnerable group in the face of climate change, women and girls are largely excluded from the formulation of policies, strategies, and programs for risk reduction and resilience. Thus, community engagement integrating women's voices in confronting the climate crisis is of utmost importance, and it becomes necessary to implement actions for risk mitigation and public awareness, targeting the population, managers, health professionals, and governments, that enable comprehensive responses to address the multifaceted impacts of this public health problem.

Health professionals play a central role in mitigating and adapting to the impacts of climate change on sexual and reproductive health. Among their responsibilities are monitoring vulnerable populations and tracking women's health outcomes related to extreme climatic events, infectious diseases, food insecurity, and situations of violence. In addition, they can work in health education, guiding women and their families on preventive measures and health-promotion practices adapted to the environmental context, and in implementing hospital and community disaster protocols, according to national and international recommendations, ensuring continuity of care related to sexual and reproductive health in crisis situations.

In this context, it is worth asking: what can health professionals do to address the risks and impacts of climate change on women's sexual and reproductive health?

FIGO (International Federation of Gynecology and Obstetrics) recognizes that the climate crisis is a global emergency and that gynecologists and obstetricians need to assume a leadership role in education, advocacy, and the production of evidence, responding to emerging consequences for health and promoting the necessary awareness.^(12)^

Additionally, these professionals can collaborate to strengthen community resilience by coordinating local networks of health, social assistance, and protection, and by promoting gender equity through reducing inequalities in access to services and resources. They also play a strategic role in the production of scientific evidence on the effects of climate change on health, providing inputs for public policies and data-driven strategies that integrate this theme and climate adaptation.

Likewise, health services need to organize themselves to adopt strategies that make clinical practices more sustainable and minimize environmental impacts.^(19)^ The American College of Obstetricians and Gynecologists^(20)^ highlights the importance of disaster preparedness for obstetric services, recommending the creation of specific designations and the standardization of communication in emergency situations, promoting integration among different institutions and regions. In addition, it emphasizes the need for structured protocols aimed at minimizing risks, ensuring continuity of care, and strengthening the resilience of health services during crises.

The climate emergency, the disasters it triggers, and the responses to confront it must be addressed from the perspective of environmental justice, a gender focus, and human rights. It is necessary that climate policies include interventions that address sexual and reproductive health as fundamental aspects to promote the well-being of girls and women around the world.^(12)^ As an example, we can highlight the actions carried out in the state of Rio Grande do Sul, Brazil, during the 2024 floods, with specific measures for the distribution of medications, including contraceptives, and the restructuring of shelters to reduce the risk of violence against women and girls. In addition, instructional technology was produced within the scope of the Federal University of Rio Grande do Sul (UFRGS), in the form of a toolkit,^(13)^ to guide maternal-and-child health care inside the installed shelters, considering the priorities of this population.

Given the above, professionals working in gynecology and obstetrics play a central role in implementing guidelines, ensuring safe care, adequate monitoring of girls and women, and effective coordination of clinical responses to disasters caused by extreme climatic events.
